# Quaternary Ammonium Salt-Functionalized PA6-Based Elastomer as an Efficient Antistatic Additive for Polypropylene

**DOI:** 10.3390/polym18172072

**Published:** 2026-08-26

**Authors:** Jia-Hao Wang, Ze-Yong Zhao, Yu-Zhong Wang

**Affiliations:** The Collaborative Innovation Center for Eco-Friendly and Fire-Safety Polymeric Materials (MoE), National Engineering Laboratory of Eco-Friendly Polymeric Materials (Sichuan), College of Chemistry, National Key Laboratory of Advanced Polymer Materials, Sichuan University, Chengdu 610064, China; wangjiahao5@stu.scu.edu.cn

**Keywords:** quaternary ammonium salts, polypropylene, thermoplastic polyamide elastomers, antistatic properties

## Abstract

Polymeric antistatic additives offer improved resistance to migration compared with low-molecular-weight agents, but high loadings are generally required to establish effective charge-dissipation pathways in nonpolar polypropylene (PP). Herein, a series of quaternary ammonium salt-functionalized polyamide 6/polyethylene glycol elastomers (QASPA6PEG) was synthesized by melt copolymerization and used as multifunctional antistatic additives for PP. Increasing the nominal QAS content decreased the surface resistivity of the elastomers from 3.24 × 10^9^ Ω to 9.71 × 10^8^ Ω. The elastomers were subsequently melt-blended with PP at loadings of 10–20 wt% using maleic-anhydride-grafted polypropylene as a compatibilizer. The surface resistivity of the blends decreased with increasing QAS content and elastomer loading, consistent with the formation of increasingly interconnected ion-conducting domains. The blend containing 20 wt% 0.4QASPA6PEG exhibited surface resistivities of 3.64 × 10^11^ Ω and 4.69 × 10^10^ Ω on days 0 and 60, respectively. Its saturated water absorption reached 4.38%, compared with 0.27% for neat PP, supporting a moisture-assisted ionic conduction mechanism. The measured bromine content remained nearly unchanged after 60 days of storage, indicating limited loss of the QAS-containing component. In addition to improving charge dissipation, QASPA6PEG enhanced the ductility and impact resistance of PP. At a loading of 20 wt%, 0.4QASPA6PEG increased the elongation at break from 358 ± 23% to 690 ± 81% and the notched impact strength from 3.16 ± 0.37 to 4.93 ± 0.45 kJm^−2^. These results demonstrate that covalently introducing ionic structures into PA6/PEG elastomers is an effective strategy for coupling antistatic modification with toughening in PP.

## 1. Introduction

Polypropylene (PP) is extensively used in packaging, household appliances, automotive components, electrical products, and construction materials because of its low density, chemical resistance, favorable mechanical properties, and convenient melt processability [1,2,3]. However, the nonpolar structure and low moisture uptake of PP result in high electrical resistivity and a pronounced tendency to accumulate static charge. Electrostatic accumulation can cause dust adsorption, handling difficulties, electrostatic discharge, malfunction of electronic components, and potential ignition hazards in flammable environments [4,5,6]. The development of effective and durable antistatic modification strategies is therefore important for extending the application of PP in environments where electrostatic charge must be controlled.

Antistatic modification of PP can be achieved by surface treatment, incorporation of conductive fillers, blending with intrinsically conductive polymers, or addition of ionic and hydrophilic compounds [7,8,9,10]. Surface coatings can provide rapid charge dissipation but may lose functionality through abrasion or delamination. Conductive fillers, such as carbon black, carbon nanotubes, and graphene, can substantially reduce electrical resistivity once a conductive network is formed; however, they may alter the color, rheological behavior, processability, and mechanical properties of PP [11,12]. The internal incorporation of antistatic additives is therefore an attractive approach because it is compatible with conventional melt processing and can be applied to components with complex geometries [13].

Low-molecular-weight antistatic agents generally migrate toward the polymer surface, where their polar or ionic groups promote moisture adsorption and charge dissipation [14,15,16]. Although such migration may generate an antistatic surface at relatively low additive contents, continuous blooming, volatilization, extraction, or removal during cleaning can lead to progressive performance loss and surface contamination. Polymeric antistatic additives exhibit lower molecular mobility and improved resistance to depletion and are consequently more suitable for applications requiring sustained antistatic behavior [17,18,19]. Nevertheless, because their mobility is restricted and they must form continuous or near-continuous charge-transport pathways within an insulating matrix, polymeric antistatic additives often require relatively high loadings. Such high loadings increase material cost and may compromise the stiffness, strength, dimensional stability, and processability of PP. Enhancing the intrinsic charge-transport ability of a polymeric additive is therefore essential for reducing its effective loading.

Thermoplastic polyamide elastomers (TPAEs) are an important class of polymeric antistatic materials. Their segmented structures generally consist of crystalline or semicrystalline polyamide hard segments and flexible polyether or polyester soft segments [20,21,22]. The polyamide domains provide thermal resistance and mechanical integrity, whereas the soft segments impart flexibility and can promote moisture uptake and segmental mobility. In particular, PA6/polyethylene glycol (PA6/PEG) elastomers contain amide and ether groups capable of interacting with water through hydrogen bonding. PEG is commonly incorporated as a flexible and hydrophilic soft segment in thermoplastic polyamide elastomers, where it improves chain mobility and contributes to elasticity and toughness. Moreover, the ether oxygen atoms of PEG can interact with water molecules and assist the transport of ionic species, making PEG-containing elastomers promising components for antistatic applications. The resulting hydrated polar domains can facilitate charge dissipation and make PA6/PEG elastomers promising antistatic modifiers for nonpolar polymers [17]. Their elastomeric character may also promote plastic deformation and energy dissipation, allowing antistatic modification and mechanical toughening to be achieved simultaneously.

Despite these advantages, the antistatic efficiency of PA6-based elastomers in PP is limited by two factors. First, PP is highly insulating and absorbs little moisture; consequently, a sufficiently interconnected antistatic phase must be established before long-range charge transport becomes effective. Second, the marked polarity difference between PA6-based elastomers and PP leads to phase separation and limited interfacial adhesion. Although maleic-anhydride-grafted polypropylene (PP-g-MAH) can improve interfacial interactions, substantial elastomer contents may still be required to produce effective charge-dissipation pathways. Commercial polyether-block-amide materials, such as PEBAX, are therefore frequently used at relatively high concentrations in nonpolar polymer matrices. Reducing the loading required for antistatic performance while retaining favorable mechanical properties remains an important challenge.

One strategy for improving the efficiency of polymeric antistatic additives is to introduce ionic groups directly into their macromolecular structures. Quaternary ammonium salts (QASs) contain a permanently charged ammonium cation and an associated counterion, commonly chloride or bromide [23]. The high polarity and ion density of these structures can increase moisture affinity and provide mobile counterions for ionic charge transport. QAS-containing small molecules, copolymers, and polymer blends have consequently been investigated for antistatic modification [24]. Zwitterionic modifiers containing ammonium sulfonate groups, for example, have been used to improve charge dissipation in poly(ethylene terephthalate) and PA6 fibers [25]. Ionic salts have also been incorporated into PP-based composites to reduce electrical resistivity [26]. However, direct addition of low-molecular-weight QASs may cause migration, leaching, and nonuniform surface accumulation. Their incorporation may also plasticize the polymer or adversely affect thermal stability. Immobilizing QAS-derived structures within a polymeric antistatic additive could reduce their macroscopic mobility while preserving a high concentration of ionic charge carriers. Furthermore, introducing hydroxyl-functional QAS structures during the synthesis of PA6/PEG elastomers provides a route to integrate the ionic component into the segmented macromolecular structure rather than physically mixing it with the PP matrix. This molecular design is expected to combine the ionic conduction of QAS groups, the moisture affinity of PA6/PEG segments, and the energy-dissipation capability of an elastomeric dispersed phase.

In this study, a dihydroxy-functional QAS was synthesized by quaternization of 3-dimethylamino-1,2-propanediol with 1-bromodecane. The retained hydroxyl groups allowed the QAS-derived structure to participate in the melt copolymerization of PA6/PEG elastomers. Three QASPA6PEG elastomers with different nominal QAS contents were prepared and melt-blended with PP at loadings of 10, 15, and 20 wt%, using PP-g-MAH as a compatibilizer. The molecular structures and intrinsic thermal, mechanical, and electrical properties of the elastomers were characterized. The effects of QAS content and elastomer loading on the phase morphology, crystallization behavior, thermal stability, moisture uptake, surface resistivity, tensile behavior, and impact resistance of the blends were systematically investigated.

Particular attention was paid to the relationship among ionic-group concentration, moisture absorption, elastomer-domain connectivity, and charge dissipation. The evolution of surface resistivity and bromine content over 60 days was further examined to assess the storage stability of the antistatic system. This work aims to establish a molecular-design strategy for improving the antistatic efficiency of PA6/PEG elastomers while exploiting their dispersed elastomeric phase to enhance the toughness of PP.

## 2. Materials and Methods

### 2.1. Materials

*ε*-Caprolactam (CPL, 99%), 1-bromodecane (99%), 3-dimethylamino-1,2-propanediol (98%), and tetrabutyl titanate (TBT, 99%) were purchased from Adamas Reagent Co., Ltd., Shanghai, China. Adipic acid (AA, 99.5%) and polyethylene glycol (PEG, Mw ≈ 1000 g mol^−1^, 99%) were supplied by Aladdin Reagent Co., Ltd., Shanghai, China. Ethanol and diethyl ether were obtained from Chengdu Kelong Chemical Co., Ltd., Chengdu, Sichuan, China.

Polypropylene (PP, grade T30S) was supplied by Dushanzi Petrochemical Co., Ltd., Dushanzi, Xinjiang, China. Maleic-anhydride-grafted polypropylene (PP-g-MAH) was obtained from Coace Chemical Co., Ltd., Xiamen, Fujian, China and used as a compatibilizer. Deionized water was prepared in the laboratory. All materials were used as received unless otherwise specified.

### 2.2. Synthesis of the Carboxyl-Terminated PA6 Prepolymer

A carboxyl-terminated PA6 prepolymer with an approximate molecular weight of 1500 and a melting temperature of approximately 190 °C was prepared by hydrolytic polymerization.

CPL (150 g), AA (15.96 g), and deionized water (3 g) were introduced into a polymerization vessel equipped with a mechanical stirrer and a reflux condenser. The reaction system was purged with nitrogen for 10 min and subsequently heated to 240 °C. After complete melting of the reactants, the mixture was stirred at this temperature for 5 h. The nitrogen flow rate was then increased to facilitate the removal of water from the reaction system. The synthetic route of the PA6 prepolymer is shown in Figure S1.

The resulting product was cooled to room temperature and crushed into small pieces. To remove residual monomer and water-soluble oligomers, the solid product was immersed in boiling water for 2 h. It was subsequently dried at 80 °C before use. The molecular weight of the prepolymer was determined by means of viscosity measurement, and the molecular weight was determined to be approximately 1500.

### 2.3. Synthesis of the Quaternary Ammonium Salt

The dihydroxy-functional QAS was synthesized through the quaternization reaction of 3-dimethylamino-1,2-propanediol with 1-bromodecane. The two reactants were dissolved in ethanol at a molar ratio of 1:1 and reacted at 70 °C for 12 h under a nitrogen atmosphere.

**Figure 1:**
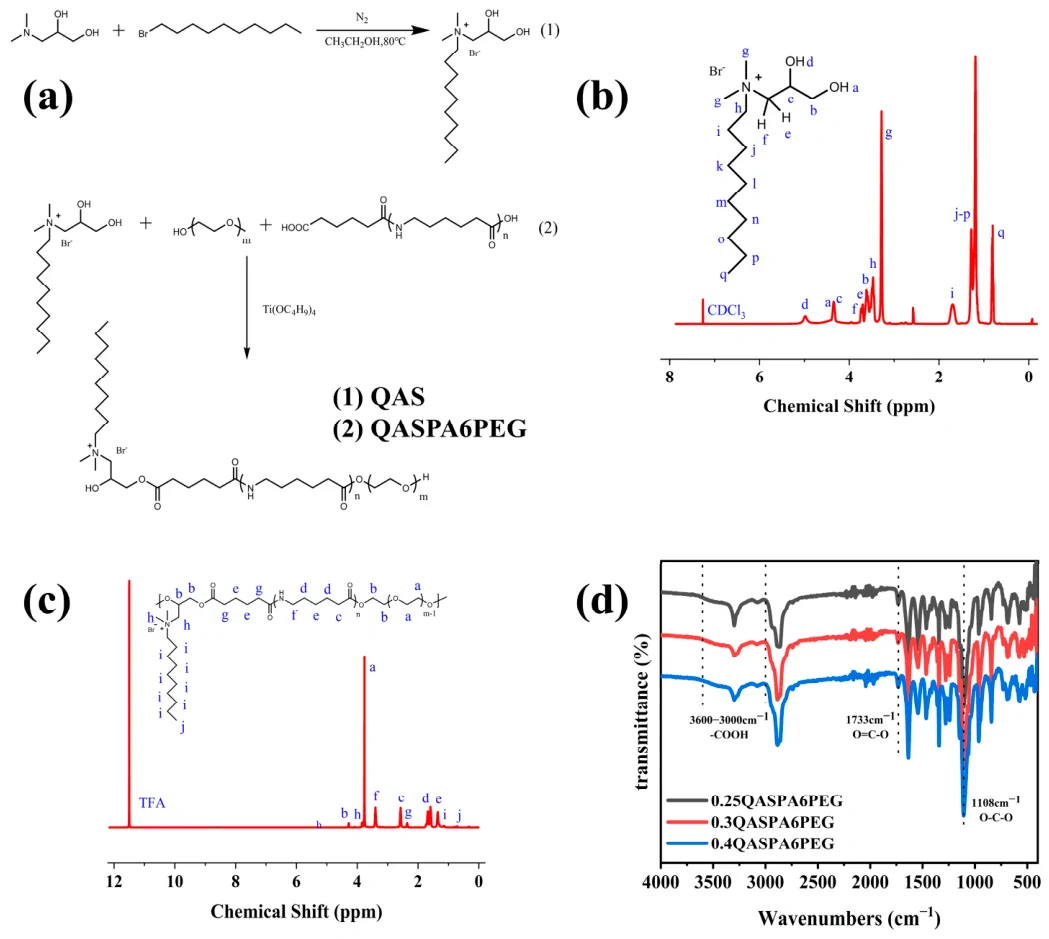
Synthesis route of QAS and QASPA6PEG (**a**), ^1^H NMR spectra of QAS (**b**), ^1^H NMR spectra of QASPA6PEG (**c**), and FTIR spectra of QASPA6PEG (**d**).

After the reaction, ethanol was removed by rotary evaporation. The product was washed three times with diethyl ether to remove residual reactants and nonionic impurities and then dried in a vacuum oven for 12 h. The resulting product was a colorless transparent liquid at room temperature. The reaction route is shown in Figure 1a.

The choice of bromide was based primarily on synthetic accessibility and reaction kinetics rather than on an expected intrinsic advantage of bromide over chloride in the antistatic performance of the final material. Chloride-containing analogues were not prepared or comparatively evaluated in the present study; therefore, no conclusion can be drawn regarding the influence of the counterion type on water absorption, phase behavior, ion mobility, or antistatic properties.

### 2.4. Synthesis of QAS-Functionalized PA6/PEG Elastomers

QAS-containing PA6/PEG elastomers were synthesized by melt copolymerization. Predetermined amounts of the PA6 prepolymer, PEG, and QAS were introduced into a polymerization vessel equipped with mechanical stirring. The reaction vessel was purged with nitrogen for 10 min and heated to 210 °C until all reactants had melted.

TBT was then introduced as the transesterification and polycondensation catalyst, and the reaction was maintained at 210 °C for 5 h. The temperature was subsequently increased to 240 °C, followed by further catalyst addition. The pressure was gradually reduced to approximately 100 Pa, and vacuum polycondensation was continued for 3–4 h.

After completion of the reaction, the products were cooled to room temperature, crushed, and dried in a vacuum oven at 80 °C for 12 h. The samples were denoted as 0.25QASPA6PEG, 0.3QASPA6PEG, and 0.4QASPA6PEG. The numerical prefix represents the nominal proportion of the PEG-based soft-segment component replaced by the QAS-containing component in the initial formulation. The detailed feed compositions are provided in Table S1, and the synthetic route is illustrated in Figure 1a.

### 2.5. Preparation of QASPA6PEG/PP Blends

QASPA6PEG/PP blends were prepared using an RM-200C torque rheometer (Harbin Hapro Electric Technology Co., Ltd., Harbin, Heilongjiang, China). The chamber temperature, rotor speed, and blending time were 200 °C, 100 rpm, and 5 min, respectively.

The QASPA6PEG contents were 10, 15, and 20 wt% of the total formulation. PP-g-MAH was maintained at 5 wt%, while the remaining component was PP. The samples were denoted according to the elastomer loading and type. For example, 20%0.4QASPA6PEG/PP refers to a blend containing 20 wt% 0.4QASPA6PEG, 5 wt% PP-g-MAH, and 75 wt% PP. The detailed feed compositions are provided in Table S2.

Commercial PEBAX/PP reference blends were prepared using the same melt-blending procedure. Their formulations and characterization results are provided in the Supplementary Materials.

### 2.6. Proton Nuclear Magnetic Resonance Spectroscopy

The ^1^H NMR spectrum of QAS was recorded using a Bruker AV II 400 MHz spectrometer with deuterated chloroform (CDCl_3_) as the solvent.

The spectra of the PA6 prepolymer, QAS-free PA6PEG, and QASPA6PEG elastomers were recorded using a Bruker AV II 600 MHz spectrometer (Bruker Corporation, Billerica, MA, USA). Deuterated trifluoroacetic acid (CF_3_COOD) was used as the solvent.

### 2.7. Fourier-Transform Infrared Spectroscopy

FTIR spectra were recorded using a Nicolet 6700 spectrometer (Thermo Fisher Scientific, Waltham, MA, USA) in reflectance mode. The spectra were collected over the range of 4000–400 cm^−1^ at a resolution of 4 cm^−1^. Each spectrum was obtained from 32 scans.

### 2.8. Solution Viscosity

The solution viscosity of the QASPA6PEG elastomers was measured according to GB/T 12006.1-2009 [27]. Polymer solutions with a concentration of 0.005 g mL^−1^ were prepared using formic acid as the solvent and tested at 25 °C using an Ubbelohde viscometer.

The viscosity number was calculated as follows:

$$[\eta ]=\left(\frac{t}{t_{0}}-1\right)\frac{1}{c}$$

where *t* is the flow time of the polymer solution, *t*_0_ is the flow time of pure formic acid, and *c* is the polymer concentration.

Because the measurements were performed at a single concentration, the results are referred to as apparent viscosity numbers rather than intrinsic viscosities extrapolated to zero concentration.

### 2.9. Tensile Testing

Tensile properties were measured using an Instron 3366 universal testing machine (Instron, Norwood, MA, USA). Dumbbell-shaped specimens were prepared using a HAAKE MiniJet Pro microinjection molding machine (Thermo Fisher Scientific, Waltham, MA, USA). The specimen gauge section was 25 mm long, 4 mm wide, and 2 mm thick.

The measurements were performed at 25 °C. The crosshead speeds were 50 mm min^−1^ for the QASPA6PEG elastomers and 10 mm min^−1^ for the PP blends. At least three specimens were tested for each formulation, and the results are reported as the mean and standard deviation.

### 2.10. Notched Impact Testing

Notched Izod impact tests were performed at 25 °C using a PJ2000 pendulum impact tester (Shenzhen Sansi Testing Instrument Co., Ltd., Shenzhen, Guangdong, China) according to GB/T 1843-2008 [28]. Rectangular specimens measuring 80 × 4 × 2 mm^3^ were prepared by microinjection molding. A V-shaped notch with a depth of 2 mm was introduced into each specimen.

At least three specimens were tested for each formulation, and the mean and standard deviation were calculated.

### 2.11. Differential Scanning Calorimetry

Differential scanning calorimetry was conducted using a DSC2500 instrument (TA Instruments, New Castle, DE, USA) under a nitrogen atmosphere. Samples weighing 3–5 mg were sealed in aluminum pans. Each sample was first heated to 250 °C at 10 °C min^−1^ to eliminate its previous thermal history. The sample was then cooled to −70 °C and reheated to 250 °C at the same rate.

The crystallization temperature, *T*_c_, was determined from the cooling scan, whereas the melting temperature, *T*_m_, and melting enthalpy were obtained from the second heating scan. The crystallinity of the PP phase was calculated using

$$X_{C}=\frac{{\Delta H}_{m}}{W\times {\Delta H}_{m}^{0}}\;\times \;100\%$$

where Δ*H*_m_ is the measured melting enthalpy assigned to PP, *w* is the mass fraction of PP in the blend, and ${\Delta H}_{m}^{0}$ = 207 Jg^−1^ is the melting enthalpy of completely crystalline PP.

### 2.12. Thermogravimetric Analysis

Thermogravimetric analysis was conducted using a TGA/DSC1/1100LF simultaneous thermal analyzer (Mettler-Toledo, Greifensee, Switzerland) under nitrogen. Samples weighing 3–5 mg were heated from 40 to 700^∘^C at a rate of 10 °C  min^−1^.

The temperature corresponding to 5% mass loss, *T*_5%_, and the temperature at the maximum mass-loss rate, *T*_max_, were obtained from the TGA and DTG curves, respectively.

### 2.13. Surface Resistivity

Surface resistivity was measured using a ZST-530 volume/surface resistivity meter (Beijing Zhonghang Times Instrument Equipment Co., Ltd., Beijing, China) according to GB/T 31838.3-2019 [29]. The specimens were prepared by compression molding using an XLB-D 400 × 400 × 3/0.50 MN flat press (Qingdao Yadong Machinery Group Co., Ltd., Qingdao, Shandong, China).

Before measurement, the specimens were conditioned at 23 °C for 24 h. The measurements were performed at 23 °C and 50% relative humidity using an applied voltage of 250 V. At least three specimens were measured for each formulation.

To evaluate storage-dependent performance, the surface resistivity was measured on days 0, 30, and 60 under the same conditioning and testing conditions.

### 2.14. Saturated Water Absorption

Saturated water absorption was measured at 23 °C according to GB/T 1034-2008 [30]. Before testing, all specimens were dried in a vacuum oven at 80 °C for 24 h. The sample mass was periodically measured using an analytical balance until no further mass change was detected.

### 2.15. Scanning Electron Microscopy and Energy-Dispersive X-Ray Spectroscopy

The fracture-surface morphology of the blends was examined using an SU3500 scanning electron microscope (Hitachi High-Tech Corporation, Tokyo, Japan). The specimens were attached to conductive adhesive tape and sputter-coated with gold before observation.

Elemental analysis was performed using an Aztec X-Max20 energy-dispersive X-ray spectroscopy system (Hitachi High-Tech Corporation, Tokyo, Japan). Bromine was used as an elemental marker for the QAS-containing phase. EDS mapping was used to evaluate its spatial distribution, and semi-quantitative EDS analysis was used to compare the measured bromine contents before and after storage.

## 3. Results and Discussion

### 3.1. Synthesis and Structural Characterization of QAS and QASPA6PEG

The synthesis route of QAS and QASPA6PEG is shown in Figure 1a. The tertiary amino group of 3-dimethylamino-1,2-propanediol reacted with 1-bromodecane through nucleophilic substitution, producing a quaternary ammonium center associated with a bromide counterion. The two hydroxyl groups in the product were retained and subsequently enabled the QAS to participate in the melt copolymerization of the PA6-based elastomer. No post-reaction extraction or purification was performed during the preparation of QASPA6PEG.

The ^1^H NMR spectrum of the synthesized QAS is shown in Figure 1b. The characteristic signals corresponding to the terminal methyl group and methylene groups of the decyl chain, the methyl groups attached to the quaternary nitrogen atom, and the hydroxyl-containing propyl structure were observed. The successful assignment of these proton signals confirmed the formation of the expected quaternized product.

In the spectrum of QASPA6PEG (Figure 1c), new peaks (δ = 3.84, 1.14 and 0.80) assigned to the QAS moiety appeared, indicating successful incorporation of the ionic component into the elastomer chain. The FTIR spectra (Figure 1d) further confirmed the expected chemical structures. The broad absorption between 3600 and 3000 cm^−1^ was associated with hydrogen-bonded N-H and O-H groups. The bands at approximately 2935 and 2870 cm^−1^ were assigned to asymmetric and symmetric stretching vibrations of aliphatic methylene groups. The absorption at approximately 1730 cm^−1^ was assigned to ester carbonyl groups formed during the reaction of hydroxyl-containing soft-segment components with carboxyl-terminated PA6 prepolymers. The band near 1105 cm^−1^ originated mainly from ether C-O-C stretching in PEG.

The intensities of the aliphatic C-H stretching bands increased with increasing nominal QAS content, consistent with the increased contribution of the decyl-containing QAS structure. Taken together, the ^1^H NMR and FTIR results support the successful incorporation of QAS-containing segments into the PA6/PEG elastomers.

### 3.2. Properties of QASPA6PEG Elastomers

The thermal, mechanical, viscosity, and electrical properties of the QASPA6PEG elastomers are summarized in Table S7.

All three elastomers exhibited tensile strengths of approximately 14–15 MPa and elongations at break between 368% and 475%, demonstrating that the segmented structures retained substantial elastomeric deformability. Increasing the nominal QAS content slightly decreased the tensile strength but increased the elongation at break. In particular, the elongation at break increased from 368 ± 12% for 0.25QASPA6PEG to 475 ± 18% for 0.4QASPA6PEG.

The increased extensibility may be associated with a higher proportion of flexible QAS-containing aliphatic structures and a reduced regularity of the hard- and soft-segment packing. Reversible electrostatic interactions between ionic groups may also contribute to energy dissipation during deformation. However, direct confirmation of this contribution would require dynamic mechanical, stress–relaxation, or cyclic tensile measurements.

The crystallization temperature decreased from 143.51 to 137.89 °C, while the melting temperature decreased from 196.38 to 190.45 °C as the QAS content increased. The QAS-containing structure was less regular than the original PA6/PEG segments and therefore interfered with chain packing and crystallization. Nevertheless, the melting temperatures remained close to or below the PP blending temperature, allowing the elastomers to be melt-processed with PP.

The *T*_5%_ values of the elastomers ranged from 334.7 to 345.0 °C, and their *T*_max_ values ranged from 393.5 to 400.0 °C. Although increasing the QAS content caused a moderate decrease in thermal stability, all elastomers remained sufficiently stable for melt blending at 200 °C.

The apparent viscosity numbers were similar for all three samples, ranging from 1.03 to 1.06 dLg^−1^. These results suggest that the change in QAS feed content did not produce a substantial difference in the solution hydrodynamic behavior of the final elastomers.

More importantly, the surface resistivity decreased from 3.24 × 10^9^ Ω for 0.25QASPA6PEG to 9.71 × 10^8^ Ω for 0.4QASPA6PEG. This trend demonstrates that the increased concentration of ionic groups and bromide counterions enhanced charge transport within the elastomer. The improved intrinsic antistatic character provided the basis for using QASPA6PEG as a polymeric antistatic additive for PP.

### 3.3. Tensile Properties of QASPA6PEG/PP Blends

**Figure 2:**
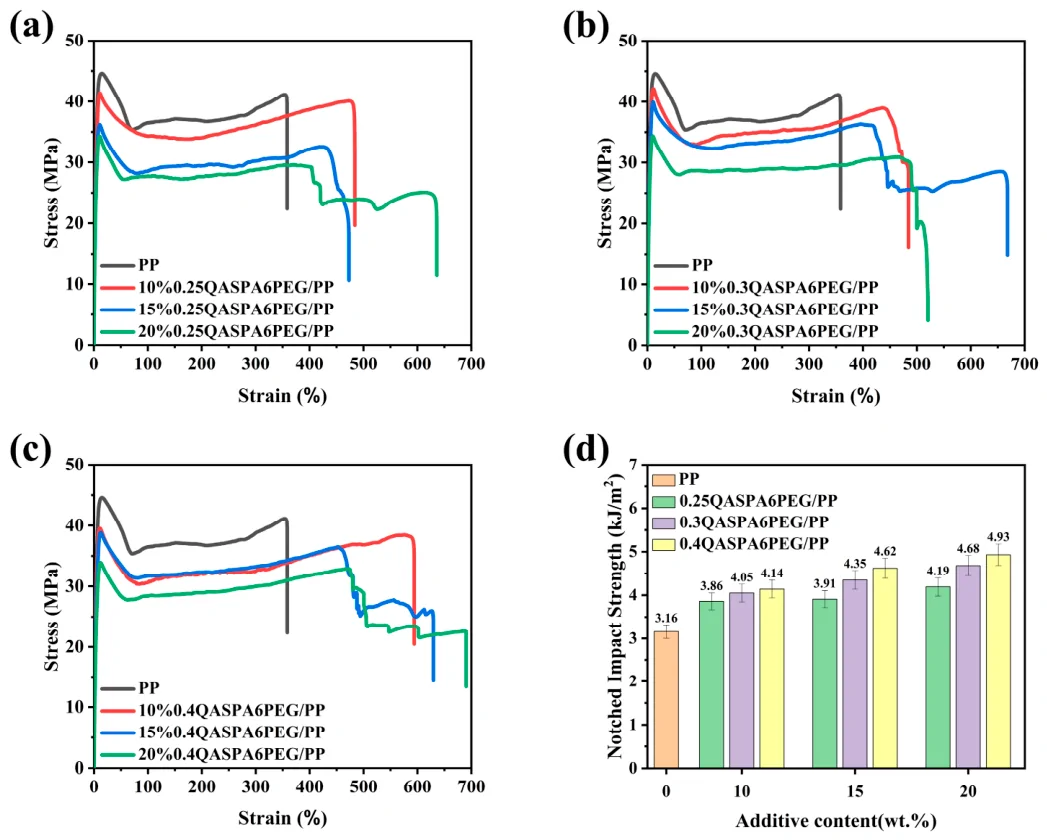
Tensile stress–strain curves of 0.25QASPA6PEG/PP (**a**), 0.3QASPA6PEG/PP (**b**), and 0.4QASPA6PEG/PP (**c**), and notched impact strength of QASPA6PEG/PP blends (**d**).

The tensile stress–strain curves and corresponding mechanical properties of the QASPA6PEG/PP blends are shown in Figure 2 and Table S10. Because all blends contained 5 wt% PP-g-MAH, its contribution to interfacial adhesion and phase morphology cannot be separated completely from that of the elastomer. The present comparisons therefore demonstrate the effect of QAS incorporation under fixed compatibilization conditions rather than the isolated effect of the QAS functionality.

The incorporation of QASPA6PEG generally decreased the Young’s modulus and yield strength of PP while increasing its elongation at break. Neat PP exhibited a Young’s modulus of 993 ± 24 MPa, a yield strength of 44.59 ± 0.62 MPa, and an elongation at break of 358 ± 23%. At an elastomer loading of 20 wt%, the Young’s modulus decreased to 709–798 MPa and the yield strength decreased to approximately 33–34 MPa. In contrast, the elongation at break increased to 635 ± 40% for 20%0.25QASPA6PEG/PP and 690 ± 81% for 20%0.4QASPA6PEG/PP.

The decrease in modulus and yield strength was primarily attributable to the lower stiffness of the elastomeric dispersed phase. Limited interfacial adhesion between the polar QASPA6PEG phase and the nonpolar PP matrix may have further reduced the efficiency of stress transfer. Although PP-g-MAH was introduced as a compatibilizer, the blends retained a phase-separated morphology, as discussed in the SEM analysis.

The increase in elongation at break indicates that QASPA6PEG promoted plastic deformation of the PP matrix. Under tensile loading, deformation of the elastomer domains and partial interfacial debonding may relieve local triaxial stress and facilitate shear yielding of the surrounding PP matrix. The flexible elastomer chains can also undergo extensive deformation and dissipate mechanical energy, thereby delaying macroscopic fracture.

At elastomer loadings of 15–20 wt%, stepwise decreases in stress were observed before final fracture in several stress–strain curves. This behavior may be associated with progressive interfacial debonding, localized neck propagation, or spatially nonuniform deformation in the multiphase blends. Instrumental factors, including grip slippage, should also be excluded before these features are assigned exclusively to a microscopic deformation mechanism.

At the same loading, increasing the nominal QAS content generally resulted in higher elongation at break, with the effect being most evident at an elastomer content of 20 wt%. This trend is consistent with the higher extensibility of the elastomers containing more QAS-derived structures. Ionic interactions may additionally contribute to energy dissipation during deformation, although their independent contribution cannot be established from the present tensile data. During tensile deformation, no obvious localized deformation or abnormal behavior was observed prior to fracture. However, examination of the fractured specimens revealed apparent skin–core delamination in both the upper and lower sections. Based on these observations, the stepwise stress drops observed in the blue and green stress–strain curves in Figure 2 may be attributed to progressive interfacial debonding and internal damage resulting from the relatively poor compatibility between the blend components.

Overall, QASPA6PEG improved the ductility of PP while the blends retained yield strengths above approximately 32 MPa. In comparison with the PEBAX/PP system (Figure S3 and Table S3 and Table S4), these findings imply that the QASPA6PEG/PP blends demonstrate a favorable balance between strength and toughness, thereby enabling them to meet the practical performance demands of conventional PP applications.

### 3.4. Notched Impact Properties

The notched impact strength increased with both QASPA6PEG loading and nominal QAS content, as shown in Figure 2d.

Neat PP exhibited a notched impact strength of 3.16 ± 0.37 kJm^−2^. The impact strength increased progressively with elastomer loading and reached 4.19 ± 0.42, 4.68 ± 0.48, and 4.93 ± 0.45 kJm^−2^ for the blends containing 20 wt% 0.25QASPA6PEG, 0.3QASPA6PEG, and 0.4QASPA6PEG, respectively. The highest value represented an increase of approximately 56% relative to neat PP.

The increased toughness may be associated with energy dissipation through deformation of the dispersed elastomer phase and plastic deformation of the PP matrix. The increased fracture-surface roughness observed by SEM was consistent with enhanced plastic deformation.

The impact results, together with the increased elongation at break, demonstrate that QASPA6PEG serves not only as an antistatic component but also as a moderate toughening agent for PP. However, the improvement in toughness was accompanied by a decrease in stiffness and yield strength. Consequently, the elastomer loading should be selected according to the required balance among antistatic performance, strength, stiffness, and toughness.

### 3.5. Melting and Crystallization Behavior

**Figure 3:**
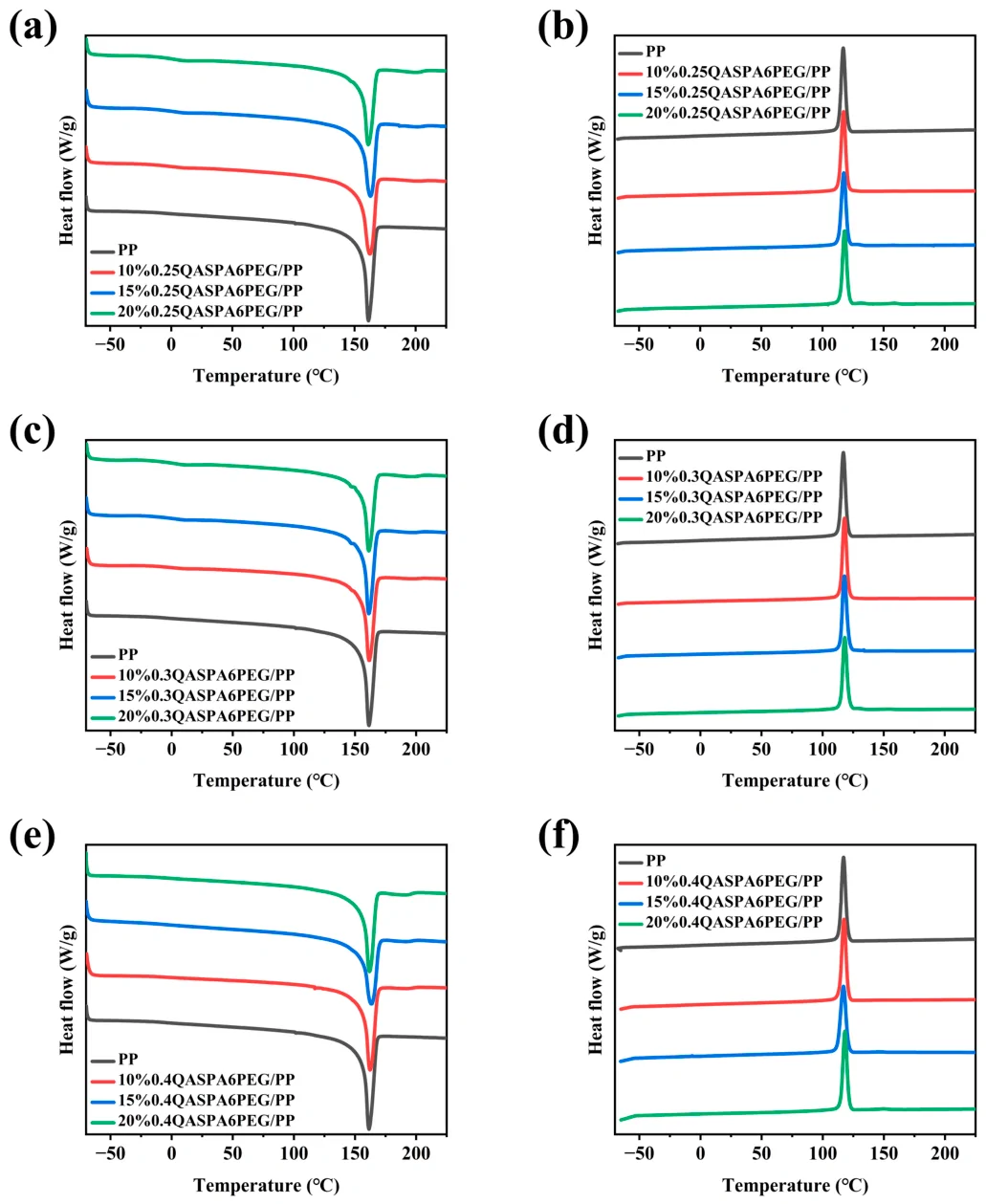
Crystallization and melting curves of QASPA6PEG/PP blends: (**a**,**b**) crystallization and melting curves, respectively, of 0.25QASPA6PEG/PP; (**c**,**d**) crystallization and melting curves, respectively, of 0.30QASPA6PEG/PP; and (**e**,**f**) crystallization and melting curves, respectively, of 0.40QASPA6PEG/PP.

The DSC cooling and second-heating curves of the blends are shown in Figure 3, and the corresponding thermal parameters are summarized in Table S9.

The melting temperatures of the blends ranged from 161.41 to 163.48 °C, compared with 161.44 °C for neat PP. Their crystallization temperatures ranged from 117.03 to 118.22 °C, compared with 117.01 °C for neat PP. These relatively small differences indicate that the incorporation of up to 20 wt% QASPA6PEG did not substantially change the principal melting and crystallization transitions of the PP phase.

After normalization to the PP content, the calculated crystallinity increased from 48.61% for neat PP to 50.27–52.93% for the blends. The moderate increase may be associated with heterogeneous nucleation at the interfaces between PP and the dispersed elastomer domains. The interfaces or certain polar components in the blends may reduce the nucleation barrier and increase the number of PP crystallization sites.

Although the impact strength and calculated crystallinity both increased after addition of QASPA6PEG, the improvement in toughness should not be attributed directly to increased crystallinity. The elastomeric dispersed phase and the associated deformation mechanisms are more likely to be the dominant causes of the enhanced impact resistance.

### 3.6. Thermal Stability

**Figure 4:**
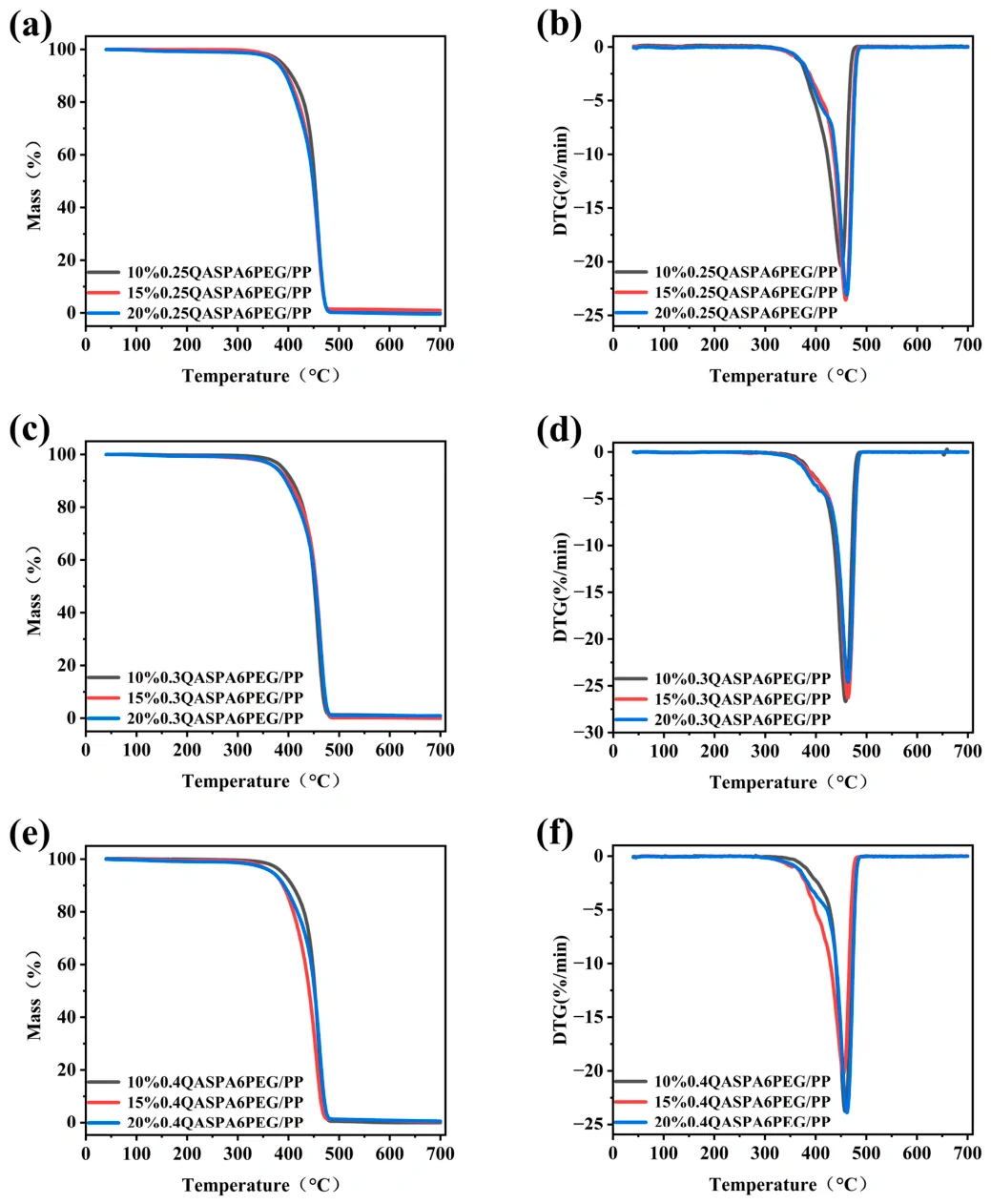
TGA/DTG curves of 0.25QASPA6PEG/PP (**a**,**b**), 0.3QASPA6PEG/PP (**c**,**d**), and 0.4QASPA6PEG/PP (**e**,**f**).

The TGA and DTG curves of the blends are shown in Figure 4, and the corresponding thermal parameters are summarized in Table S8. All formulations exhibited one dominant mass-loss stage under nitrogen, indicating that the degradation processes of PP and QASPA6PEG overlapped within the measured temperature range.

Neat PP exhibited a *T*_5%_ of 333.6 °C and a *T*_max_ of 408.1 °C. After incorporation of QASPA6PEG and PP-g-MAH, the *T*_5%_ values of the blends ranged from 368.3 to 389.3 °C, while their *T*_max_ values ranged from 430.0 to 463.3 °C.

The measured degradation temperatures therefore did not decrease after addition of QASPA6PEG. The apparent shift toward higher temperatures may arise from intermolecular interactions among PP, PP-g-MAH, and the PA6-based elastomer, as well as changes in heat and mass transfer caused by the multiphase structure. The polar polyamide-rich phase may also influence the diffusion of volatile degradation products.

Regardless of the detailed stabilization mechanism, all blends exhibited *T*_5%_ values at least approximately 168 °C above the melt-blending temperature of 200 °C. Therefore, the QASPA6PEG/PP blends possessed sufficient thermal stability for conventional PP melt processing.

### 3.7. Surface Resistivity and Storage-Dependent Antistatic Performance

The surface resistivity of the blends was measured at 23 °C and 50% relative humidity. Under the classification criterion adopted in this study, materials with surface resistivities below 10^12^ Ω were considered to exhibit antistatic behavior.

**Figure 5:**
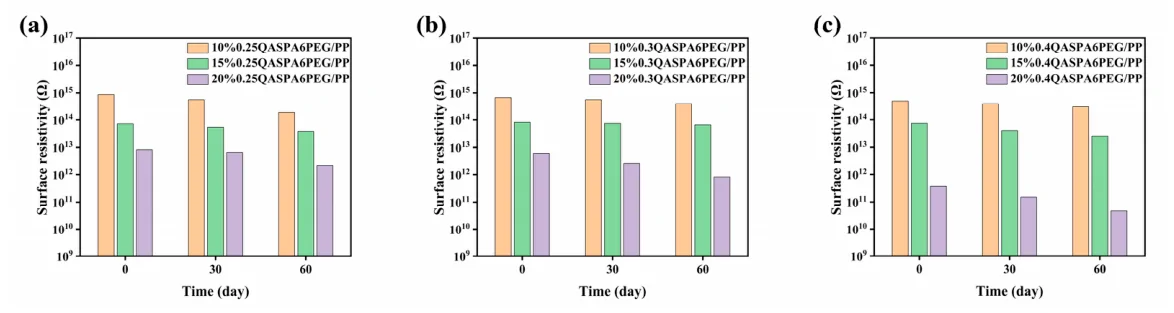
Surface resistivity of 0.25QASPA6PEG/PP (**a**), 0.3QASPA6PEG/PP (**b**), and 0.4QASPA6PEG/PP (**c**).

As shown in Figure 5, the surface resistivity decreased with increasing QASPA6PEG loading. At low elastomer contents, the QASPA6PEG domains were predominantly isolated within the electrically insulating PP matrix. The large interdomain distance limited charge transfer, and the blends therefore retained relatively high surface resistivity.

Increasing the elastomer content reduced the distance between the polar and ionic domains and promoted the formation of more continuous charge-dissipation pathways. A particularly pronounced reduction in resistivity occurred at 20 wt% elastomer loading, indicating that the system approached a composition at which the QASPA6PEG-rich domains became sufficiently interconnected for effective charge transport.

At a given elastomer loading, the surface resistivity generally decreased with increasing QAS content. On day 0, the blends containing 20 wt% 0.25QASPA6PEG, 0.3QASPA6PEG, and 0.4QASPA6PEG exhibited resistivities of 8.15 × 10^12^, 6.11 × 10^12^, and 3.64 × 10^11^ Ω, respectively. Only the 20%0.4QASPA6PEG/PP blend was below 10^12^ Ω immediately after preparation and conditioning.

The enhanced performance at higher QAS contents was attributed to the increased density of quaternary ammonium groups and bromide counterions. These ionic species increased the number of charge carriers and promoted ionic transport through the hydrophilic elastomer domains.

The surface resistivity of most blends decreased during storage rather than increasing. For example, the surface resistivity of 20%0.4QASPA6PEG/PP decreased from 3.64 × 10^11^ Ω on day 0 to 1.49 × 10^11^ Ω on day 30 and 4.69 × 10^10^ Ω on day 60. Similarly, 20%0.3QASPA6PEG/PP reached 8.15 × 10^11^ Ω after 60 days.

The storage-dependent decrease may be associated with gradual equilibration of the hydrophilic QASPA6PEG domains with environmental moisture. Slow structural rearrangement or redistribution of the elastomer-rich phase near the specimen surface may also improve the continuity of charge-dissipation pathways. These processes differ from simple depletion of an antistatic component because the measured bromine content remained nearly unchanged during the same period.

Compared under the same processing and testing conditions, 20 wt% 0.4QASPA6PEG provided antistatic performance comparable to that obtained using approximately 25 wt% commercial PEBAX (shown in Table S6). This result suggests that QAS functionalization improved the additive efficiency of the PA6-based elastomer. The absence of surface-resistivity measurements after saturated water absorption has been acknowledged as a limitation of the present study. However, the lack of surface-resistivity measurements after saturated water absorption is acknowledged as a limitation of the present study.

### 3.8. Saturated Water Absorption and Moisture-Assisted Ionic Conduction

**Figure 6:**
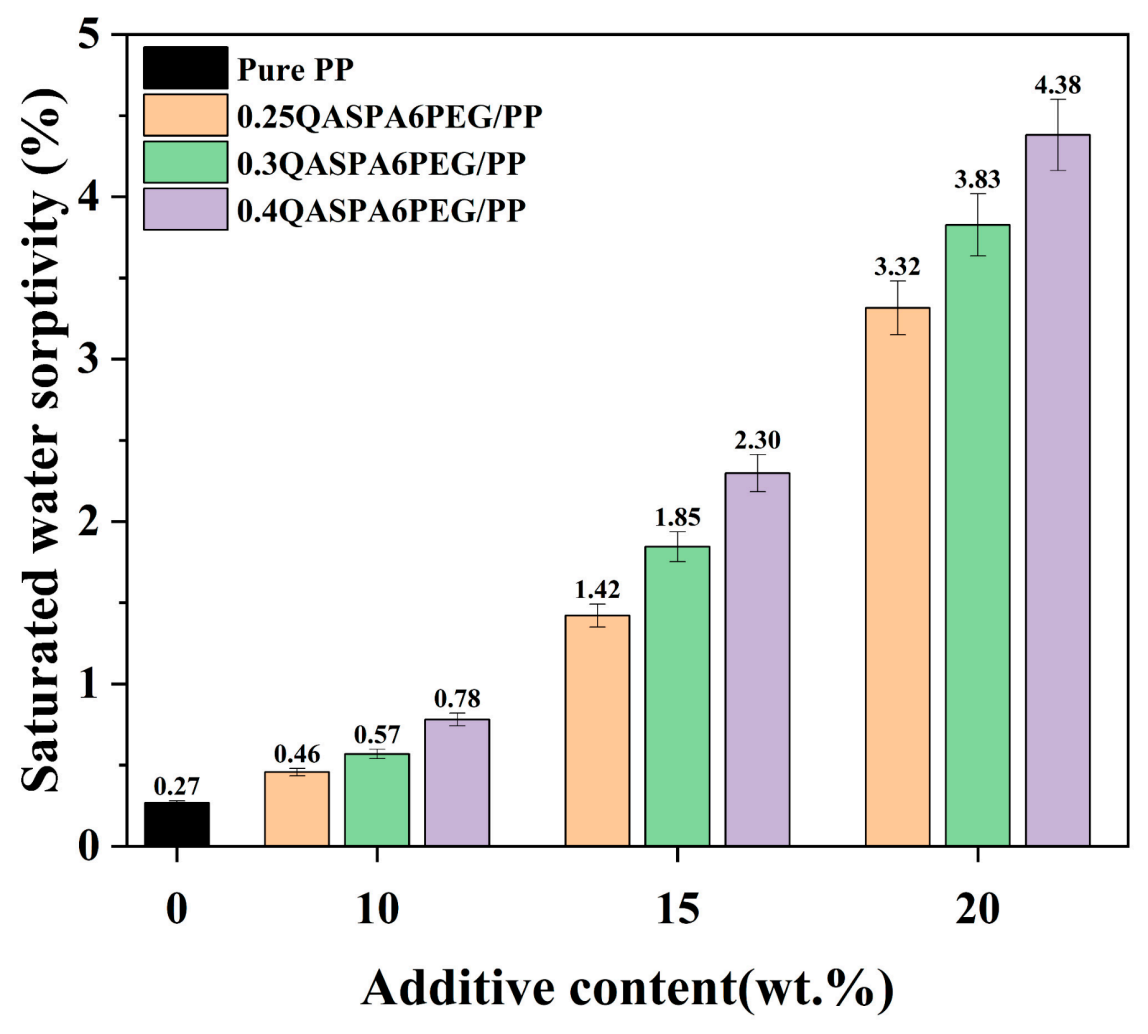
Saturated water absorption of QASPA6PEG/PP blends.

The saturated water absorption of QASPA6PEG/PP blends is shown in Figure 6. Neat PP exhibited a saturated water absorption of only 0.27%, consistent with its nonpolar structure. The saturated water absorption increased with increasing QASPA6PEG loading and nominal QAS content. The blend containing 20 wt% 0.4QASPA6PEG exhibited the highest measured value of 4.38%, corresponding to approximately 16 times the value of neat PP.

The increased water absorption was attributed to the combined contributions of the ether groups in PEG, amide groups in PA6, hydroxyl-containing QAS structures, and quaternary ammonium/bromide ion pairs. These polar and ionic groups interact with water through hydrogen bonding, ion–dipole interactions, and hydration of the ionic species.

Adsorbed water can facilitate charge dissipation through two complementary effects. First, hydration of the quaternary ammonium groups and bromide counterions can increase the mobility of ionic charge carriers within the QASPA6PEG-rich phase. Second, moisture adsorption at or near the specimen surface can create a more conductive interfacial layer through which accumulated electrostatic charge can dissipate.

Based on the water-absorption, surface-resistivity, and morphological results, the antistatic behavior of the blends was attributed to the combined effects of (i) ionic charge transport involving quaternary ammonium groups and bromide counterions, (ii) moisture uptake by the polar PA6-, PEG-, and QAS-containing structures, (iii) enhanced ionic mobility within hydrated polar domains, and (iv) increased connectivity among QASPA6PEG-rich domains at sufficiently high elastomer loadings.

### 3.9. Phase Morphology

**Figure 7:**
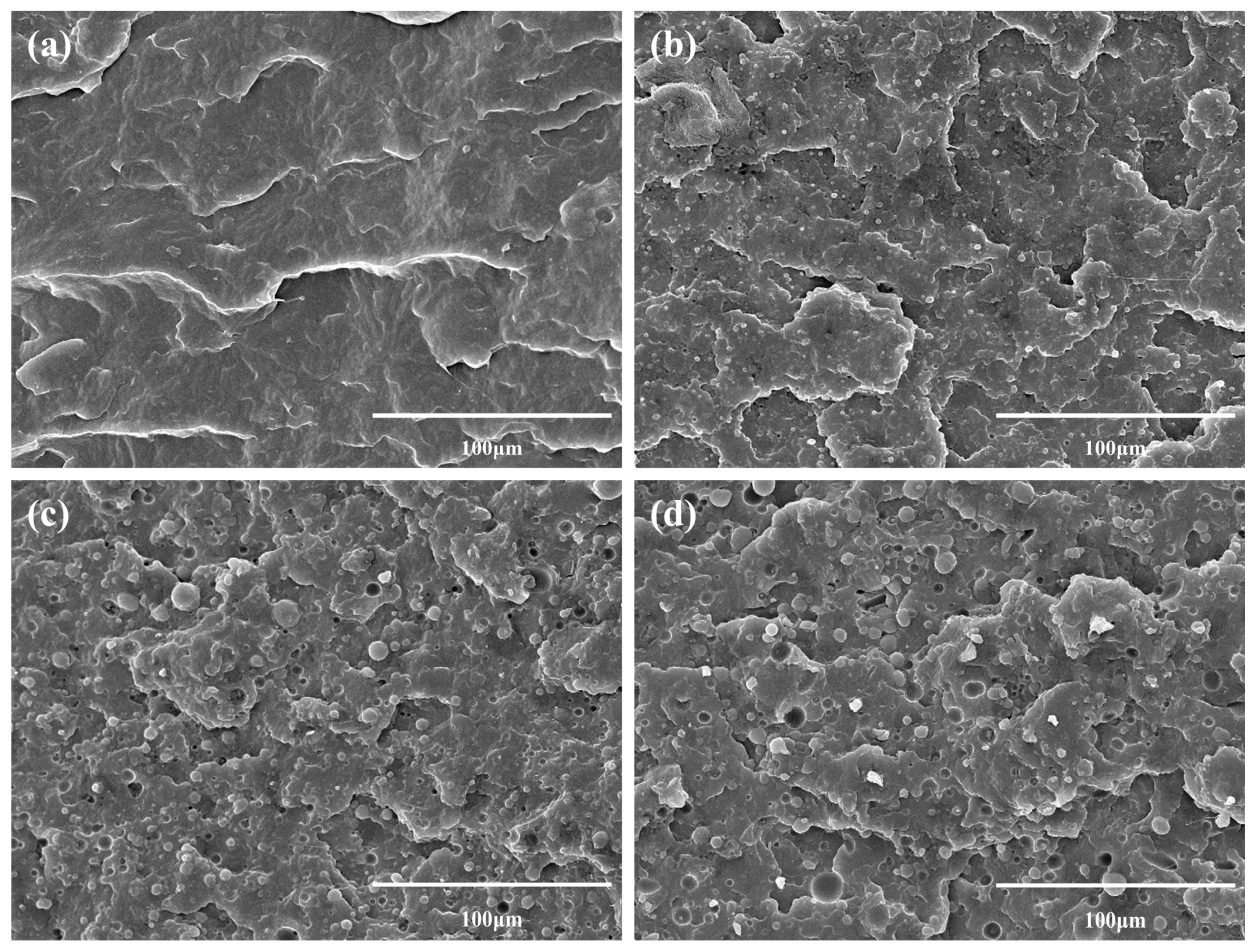
SEM images of QASPA6PEG/PP: (**a**) pure PP, (**b**) 10%0.4QASPA6PEG/PP, (**c**) 15%0.4QASPA6PEG/PP, and (**d**) 20%0.4QASPA6PEG/PP.

The fracture-surface morphologies of neat PP and the 0.40QASPA6PEG/PP blends are shown in Figure 7. Neat PP exhibited a comparatively smooth fracture surface. After the incorporation of QASPA6PEG, the fracture surfaces became rougher and exhibited dispersed domains, interfacial cavities, and locally elongated deformation features. These morphological changes became more pronounced with increasing elastomer loading and were qualitatively consistent with the increases in elongation at break and notched impact strength.

The blends displayed a typical sea–island morphology, in which the QASPA6PEG-rich phase was dispersed within the continuous PP matrix. This morphology confirms that the polar QASPA6PEG elastomer and nonpolar PP matrix remained phase-separated under the processing conditions employed. As the elastomer content increased, the apparent number density and spatial coverage of the dispersed domains increased, while the average separation between neighboring domains decreased. Local aggregation and interfacial cavities were also observed at higher elastomer loadings.

The evolution of the phase morphology was relevant to both the mechanical and electrical properties of the blends. Under mechanical loading, deformation of the elastomer-rich domains and partial interfacial debonding may relieve local stress concentrations and promote plastic deformation of the surrounding PP matrix. These processes provide a plausible explanation for the increased elongation at break and impact strength, although direct confirmation of domain cavitation and matrix shear yielding would require microscopic examination of specimens deformed under controlled tensile or impact conditions.

The decreasing distance between neighboring QASPA6PEG-rich domains may also facilitate charge dissipation. At low elastomer contents, the ionic domains were predominantly separated by the electrically insulating PP matrix. At higher loadings, the increased domain density and closer domain–domain proximity were more favorable for charge transfer through interconnected or near-interconnected polar regions. The morphological observations are therefore consistent with the proposed formation of continuous charge-dissipation pathways at high QASPA6PEG loadings.

### 3.10. Bromine Distribution and Retention

Bromine was used as an elemental marker for the QAS-containing phase because it originated from the bromide counterion of the synthesized QAS. The EDS elemental mapping of bromine in 20% 0.4QASPA6PEG/PP and the bromine contents of the QASPA6PEG/PP composites are presented in Figure S4 and Table S11, respectively. The EDS maps showed that bromine was broadly distributed throughout the analyzed regions of the blends. This result indicates that the QAS-containing elastomer was dispersed across the PP matrix rather than being confined to a limited number of large agglomerates.

The initial measured bromine contents of the QASPA6PEG elastomers were close to their theoretical values. The measured-to-theoretical ratios were approximately 94–96%, suggesting high retention of the bromine-containing QAS component during synthesis, purification, and processing.

The bromine contents of the blends changed only slightly after 60 days of storage. For example, 20%0.4QASPA6PEG/PP exhibited a theoretical bromine content of 0.57% and an initial measured content of 0.54%, which remained at 0.54% after 60 days. Similarly small changes were observed for the other formulations.

These results indicate limited depletion of the bromine-containing ionic component from the analyzed bulk regions during storage. The stable bromine content, together with the continued decrease in surface resistivity, supports the storage durability expected for a polymeric antistatic additive.

## 4. Conclusions

A series of quaternary-ammonium-salt-functionalized PA6/PEG elastomers (QASPA6PEG) was synthesized by melt copolymerization and evaluated as polymeric antistatic and toughening additives for polypropylene (PP). The ^1^H NMR and FTIR results verified the formation of the synthesized QAS and were consistent with the incorporation of QAS-containing structures into the PA6/PEG elastomers. Increasing the nominal QAS content improved the electrical conductivity of the elastomers, as indicated by a decrease in surface resistivity from 3.24 × 10^9^ to 9.71 × 10^8^ Ω. The elastomers also exhibited tensile strengths of approximately 14–15 MPa, elongations at break of 368–475%, and sufficient thermal stability for the melt-processing conditions used in this study.

The incorporation of QASPA6PEG into PP reduced Young’s modulus and yield strength but increased elongation at break and notched impact strength. Among the investigated formulations, the blend containing 20 wt% 0.40QASPA6PEG exhibited the best combined antistatic and toughening performance. Its elongation at break and notched impact strength reached 690 ± 81% and 4.93 ± 0.45 kJm^−2^, respectively, compared with 358 ± 23% and 3.16 ± 0.37 kJm^−2^ for neat PP. These results demonstrate that QASPA6PEG functioned as both a charge-dissipating additive and an elastomeric toughening phase, although the improvements in ductility and impact resistance were accompanied by reductions in stiffness and yield strength.

The addition of QASPA6PEG caused only minor changes in the melting and crystallization temperatures of PP. All blends exhibited 5% mass-loss temperatures above 368 °C under nitrogen, confirming that no pronounced thermal degradation occurred within the processing temperature range employed. Surface resistivity decreased with increasing elastomer loading and nominal QAS content. The surface resistivity of the 20 wt% 0.40QASPA6PEG/PP blend decreased from 3.64 × 10^11^ Ω on day 0 to 4.69 × 10^10^ Ω after 60 days of storage. Its saturated water absorption reached 4.38%, approximately 16 times that of neat PP.

The combined electrical, water-absorption, and morphological results were consistent with a moisture-assisted ionic conduction mechanism. Increasing the QASPA6PEG loading reduced the separation between the elastomer-rich domains and may have produced closely spaced or partially interconnected charge-dissipation pathways. Moisture uptake by the polar and ionic structures likely increased counterion mobility and facilitated charge transfer within the elastomer-rich phase and near the specimen surface. Nevertheless, further impedance or dielectric spectroscopy measurements and conductivity tests under controlled-humidity conditions are required to determine the dominant conduction mechanism.

SEM observations revealed a phase-separated sea–island morphology, with increasing spatial coverage and decreasing separation of the QASPA6PEG-rich domains at higher elastomer loadings. This morphological evolution was consistent with both improved charge dissipation and increased mechanical energy absorption. EDS mapping detected bromine across the analyzed regions, and the measured bromine contents before and after 60 days of storage were comparable within the semi-quantitative accuracy of the technique. These findings indicate that no pronounced depletion of the bromine-containing component was detected from the analyzed regions over the investigated storage period. Together with the retention of the electrical performance, these results support the short-term storage stability of the polymeric antistatic system under the conditions examined.

Overall, QAS functionalization is a promising strategy for improving the charge-dissipation capability of PA6/PEG elastomers while simultaneously enhancing the ductility and impact resistance of PP.

## Data Availability

Data is contained within the article or supplementary material: The original contributions presented in this study are included in the article/Supplementary Material. Further inquiries can be directed to the corresponding author.

## Supplementary Materials

The following supporting information can be downloaded at: https://www.mdpi.com/article/10.3390/polym18172072/s1. Figure S1. Synthesis Steps of PA6 Prepolymer; Figure S2. TG (a) and DTG (b) curves of pure PP. Figure S3. The tensile stress-strain curves of PEBAX/PP; Figure S4. Bromine of EDS diagrams in 20%0.4QASPA6PEG/PP; Table S1. The composition of QASPA6PEG; Table S2. The composition of QASPA6PEG/PP blending materials; Table S3. The composition of PEBAX/PP blending materials; Table S4. Tensile data of PEBAX/PP; Table S5. The notched impact strength of PEBAX/PP; Table S6. Surface resistivity of PEBAX/PP; Table S7. The characters of QASPA6PEG; Table S8. TG data of QASPA6PEG/PP; Table S9. The melting point (Tm), crystallizing point (Tc) and crystallinity (Xc) of QASPA6PEG/PP; Table S10. Tensile data of QASPA6PEG/PP; Table S11. Bromine content in QASPA6PEG/PP.
