# Supplementary material for: Quaternary Ammonium Salt-Functionalized PA6-Based Elastomer as an Efficient Antistatic Additive for Polypropylene
Source: Polymers (Basel). 2026 Aug 26;18(17):2072. doi: 10.3390/polym18172072 (PMC13567964; doi:10.3390/polym18172072)
Supplement: Supplementary file 1 [file polymers-18-02072-s001.zip › polymers-4505129-supplementary.pdf]

Quaternary ammonium salt-functionalized PA6-based elastomer as an efficient antistatic additive for polypropylene

Jia-Hao Wang, Ze-Yong Zhao * and Yu-Zhong Wang *

The Collaborative Innovation Center for Eco-Friendly and Fire-Safety Polymeric Materials (MoE), National Engineering Laboratory of Eco-Friendly Polymeric Materials (Sichuan), College of Chemistry, National Key Laboratory of Advanced Polymer Materials, , Sichuan University,
Chengdu 610064, China;

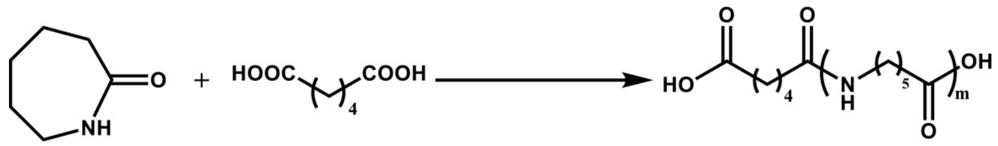

Figure S1. Synthesis Steps of PA6 Prepolymer.

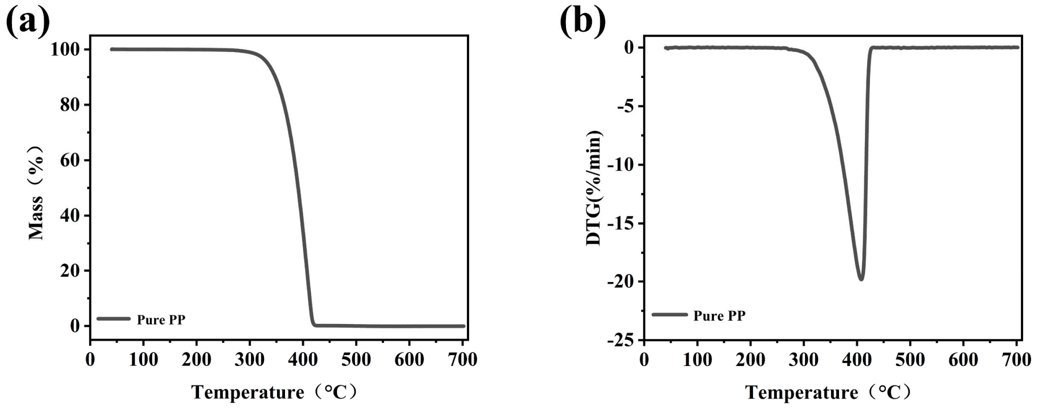

Figure S2. TG (a) and DTG (b) curves of pure PP.

Table S1. The composition of QASPA6PEG.

PrePA6(g)	PEG(g)	QAS(g)	Sample name
30	15	1.70	0.25QASPA6PEG
30	14	2.04	0.3QASPA6PEG
30	12	2.72	0.4QASPA6PEG

Table S2. The composition of QASPA6PEG/PP blending materials.

Types of elastomers	Mass of elastomer(g)	Compatibilizer(g)	PP(g)	Sample name
0.25QASPA6PEG	4	2	34	10%0.25QASPA6PEG /PP
0.25QASPA6PEG	6	2	32	15%0.25QASPA6PEG /PP
0.25QASPA6PEG	8	2	30	20%0.25QASPA6PEG /PP
0.3QASPA6PEG	4	2	34	10%0.3QASPA6PEG /PP
0.3QASPA6PEG	6	2	32	15%0.3QASPA6PEG /PP
0.3QASPA6PEG	8	2	30	20%0.3QASPA6PEG /PP
0.4QASPA6PEG	4	2	34	10%0.4QASPA6PEG /PP
0.4QASPA6PEG	6	2	32	15%0.4QASPA6PEG /PP

0.4QASPA6PEG	8	2	30	20%0.4QASPA6PEG<br>/PP

Table S3. The composition of PEBAX/PP blending materials.

Types of elastomers	Mass of elastomer(g)	Compatibilizer(g)	PP(g)	Sample name
PEBAX	6	2	32	15%PEBAX/PP
PEBAX	8	2	30	20%PEBAX/PP
PEBAX	10	2	28	25%PEBAX/PP

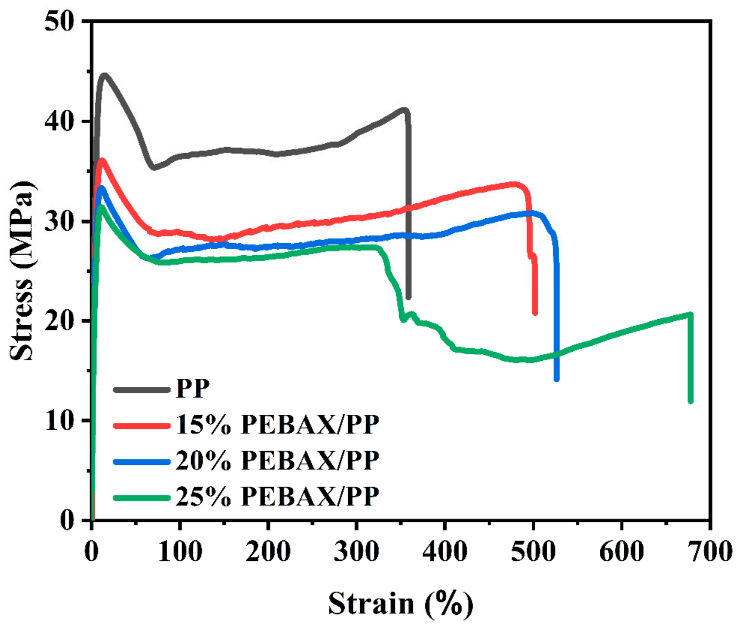

Figure S3. The tensile stress-strain curves of PEBAX/PP.

Table S4. Tensile data of PEBAX/PP.

Sample name	Young's modulus (MPa)	Elongation at break (%)	Yield strength (MPa)
PP	993±24	358±23	44.59±0.62
15%PEBAX/PP	852±57	501±50	36.08±0.85

20%PEBAX/PP	785±34	526±34	33.34±1.37
25%PEBAX/PP	686±38	677±75	31.43±0.78

Table S5. The notched impact strength of PEBAX/PP.

Sample name	Notched impact strength (kJ/m^2)
PP	3.16±0.37
15%PEBAX/PP	3.66±0.52
20%PEBAX/PP	4.94±0.49
25%PEBAX/PP	5.71±0.83

Table S6. Surface resistivity of PEBAX/PP.

Sample name	Surface resistivity (Ω) on day 0
PP	8.754×10^{15}
15%PEBAX/PP	5.947×10^{13}
20%PEBAX/PP	2.261×10^{12}
25%PEBAX/PP	2.875×10^{11}

Table S7. The characters of QASPA6PEG.

Sample	T_c (℃)	T_m (℃)	$T_{5\%}$ (℃)	T_{max} (℃)	Tensile Strength (MPa)	Elongation at break (%)	[η] (dL/g)	Surface resistivity (Ω)
0.25QASPA6PEG	143.51	196.38	345.0	400.0	15.32±0.81	368±12	1.03	3.24×10^{9}
0.3QASPA6PEG	142.62	193.24	334.7	399.3	14.30±0.64	422±21	1.05	2.19×10^{9}

0.4QASPA6PEG	137.89	190.45	335.3	393.5	14.33±0.75	475±18	1.06	9.71×10^{8}

Table S8. TG data of QASPA6PEG/PP.

Sample name	$T_{5\%}$ (°C)	T_{max} (°C)	Residues(%)
PP	333.6	408.1	#
10%0.25QASPA6PEG/PP	388.8	451.0	0.03
15%0.25QASPA6PEG/PP	380.5	458.8	#
20%0.25QASPA6PEG/PP	377.5	460.7	#
10%0.3QASPA6PEG/PP	388.2	458.5	0.91
15%0.3QASPA6PEG/PP	376.0	463.2	#
20%0.3QASPA6PEG/PP	374.8	463.3	0.81
10%0.4QASPA6PEG/PP	372.3	450.0	0.23
15%0.4QASPA6PEG/PP	368.8	454.8	0.02
20%0.4QASPA6PEG/PP	368.3	461.7	0.59

Table S9. The melting point (T_m), crystallizing point (T_c) and crystallinity (X_c) of QASPA6PEG/PP.

Sample name	T_m (°C)	T_c (°C)	X_c(%)
PP	161.44	117.01	48.61
10%0.25QASPA6PEG/PP	162.54	117.09	50.27
15%0.25QASPA6PEG/PP	163.17	117.14	51.43
20%0.25QASPA6PEG/PP	161.41	117.95	52.41
10%0.3QASPA6PEG/PP	161.61	117.92	50.54
15%0.3QASPA6PEG/PP	161.77	117.83	51.54
20%0.3QASPA6PEG/PP	161.59	118.07	52.62
10%0.4QASPA6PEG/PP	162.19	117.43	50.96
15%0.4QASPA6PEG/PP	163.48	117.03	51.72
20%0.4QASPA6PEG/PP	161.85	118.22	52.93

Table S10. Tensile data of QASPA6PEG/PP.

Sample name	Young's modulus (MPa)	Elongation at break (%)	Yield strength (MPa)
PP	993±24	358±23	44.59±0.62
10%0.25QASPA6PEG/PP	869±25	484±33	41.36±0.42
15%0.25QASPA6PEG/PP	832±27	473±51	36.17±1.04
20%0.25QASPA6PEG/PP	798±45	635±40	34.26±1.15
10%0.3QASPA6PEG/PP	851±26	489±31	42.14±1.61
15%0.3QASPA6PEG/PP	784±38	668±25	39.92±1.58
20%0.3QASPA6PEG/PP	722±54	521±41	34.35±1.36
10%0.4QASPA6PEG/PP	882±81	594±55	39.51±1.38
15%0.4QASPA6PEG/PP	847±43	630±71	38.79±1.48
20%0.4QASPA6PEG/PP	709±91	690±81	32.90±2.89

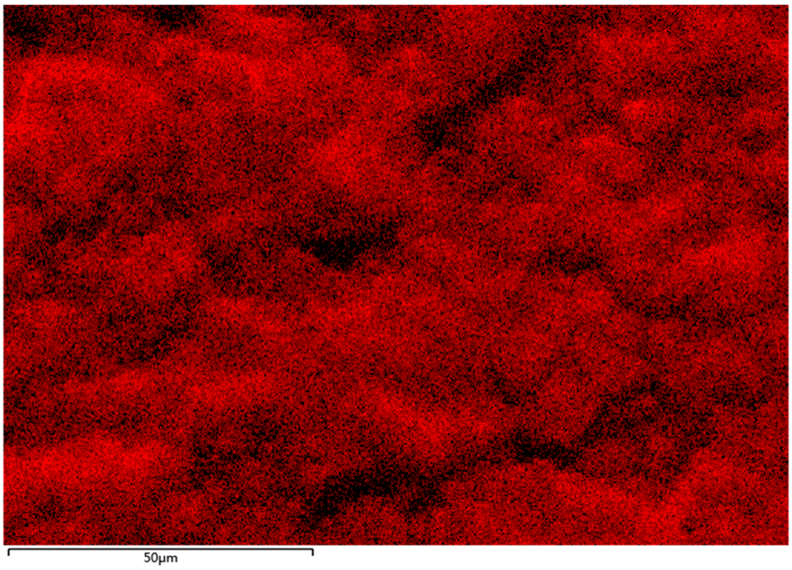

Figure S4. Bromine of EDS diagrams in 20%0.4QASPA6PEG/PP.

Table S11. Bromine content in QASPA6PEG/PP.

Sample name	Theoretical Content（%）	Actual content（%）	Actual content on day60（%）
0.25QASPA6PEG	1.71	1.62	
0.3QASPA6PEG	2.08	2.00	
0.4QASPA6PEG	2.86	2.72	
10%0.25QASPA6PEG	0.17	0.16	0.15
15%0.25QASPA6PEG	0.26	0.24	0.24
20%0.25QASPA6PEG	0.34	0.32	0.31
10%0.3QASPA6PEG	0.21	0.20	0.20
15%0.3QASPA6PEG	0.31	0.30	0.29
20%0.3QASPA6PEG	0.42	0.40	0.39
10%0.4QASPA6PEG	0.29	0.27	0.26
15%0.4QASPA6PEG	0.43	0.41	0.40
20%0.4QASPA6PEG	0.57	0.54	0.54
